# Maternal mortality in the rural Gambia, a qualitative study on access to emergency obstetric care

**DOI:** 10.1186/1742-4755-2-3

**Published:** 2005-05-04

**Authors:** Mamady Cham, Johanne Sundby, Siri Vangen

**Affiliations:** 1 Department of State for Health, Medical and Health Headquarters, Banjul, The Gambia; 2 Institute of Community Medicine, Faculty of Medicine, University of Oslo, Norway; 3 Norwegian Institute of Public Health, Oslo, Norway

## Abstract

**Background:**

Maternal mortality is the vital indicator with the greatest disparity between developed and developing countries. The challenging nature of measuring maternal mortality has made it necessary to perform an action-oriented means of gathering information on where, how and why deaths are occurring; what kinds of action are needed and have been taken. A maternal death review is an in-depth investigation of the causes and circumstances surrounding maternal deaths. The objectives of the present study were to describe the socio-cultural and health service factors associated with maternal deaths in rural Gambia.

**Methods:**

We reviewed the cases of 42 maternal deaths of women who actually tried to reach or have reached health care services. A verbal autopsy technique was applied for 32 of the cases. Key people who had witnessed any stage during the process leading to death were interviewed. Health care staff who participated in the provision of care to the deceased was also interviewed. All interviews were tape recorded and analyzed by using a *grounded theory approach*. The standard WHO definition of maternal deaths was used.

**Results:**

The length of time in delay within each phase of the model was estimated from the moment the woman, her family or health care providers realized that there was a complication until the decision to seeking or implementing care was made. The following items evolved as important: underestimation of the severity of the complication, bad experience with the health care system, delay in reaching an appropriate medical facility, lack of transportation, prolonged transportation, seeking care at more than one medical facility and delay in receiving prompt and appropriate care after reaching the hospital.

**Conclusion:**

Women do seek access to care for obstetric emergencies, but because of a variety of problems encountered, appropriate care is often delayed. Disorganized health care with lack of prompt response to emergencies is a major factor contributing to a continued high mortality rate.

## Background

Maternal mortality is the vital indicator with the greatest disparity between developed and developing countries [1,2]. Causes of maternal deaths are similar in these countries, however the distribution of causes differ somewhat from region to region [3].

Measuring maternal mortality is notoriously difficult for both conceptual and practical reasons. The currently available approaches are complex, resource intensive and imprecise; and the results they yield are often misleading [4]. The challenging nature of measuring maternal mortality necessitates to perform an action-oriented means of gathering information on where, how and why deaths are occurring; what kinds of action are needed and have been taken [5]. Assessing the impact of preventive measures demands exact knowledge about how many lives were saved. Often the answer to the "why" is not a simple one. The death may occur as a result of a series of interconnected events rather than one single factor. Answering the "why" question thus requires a systematic review of each maternal death in order to find information on events surrounding the deaths. A maternal death review is an in-depth investigation of the causes and circumstances surrounding maternal deaths such as problems in accessing care, mismanagement and inadequate routines [6]. Levels of maternal mortality in the Gambia are unacceptably high and are ranked among the highest in Africa, estimated at 1,050 per 100,000 live births and are higher in rural than in urban areas [7]. Obstetric causes of maternal deaths in the Gambia are well documented [7-11], but little attention is paid to the contributing factors. In order to reach the Millennium Development Goal of reducing maternal mortality, women's access to good quality health care embedded in a human rights framework is an important factor. Access to emergency obstetric care and better social status of women are two elements that may contribute significantly towards this goal. Site-specific information may be a key to policy change and action, even if the general causes of continued high maternal mortality rates are well known. The influence of a local maternal care access study on improving service delivery and organization has been demonstrated in Mali [12].

The objectives of the present study were to describe the socio-cultural and health service factors associated with maternal deaths in rural Gambia. We aimed at identifying factors that if avoided, may have prevented these deaths. A key focus of the study is to try to create an operative understanding of the concept of access. The study was carried out in Central and Upper River Divisions between January and September 2002. The main qualitative findings from verbal autopsies performed on 32 maternal deaths are presented. The analysis of socio-demographic background, medical causes and management of cases is presented in another paper [13].

### Study Area

Central and Upper River Divisions are located at least 300 km away from the capital city, Banjul, and the roads are generally in poor condition. In 2002 the total population of the two divisions was estimated at 400,917 [14]. The inhabitants are mainly subsistence farmers, and they are generally poor.

There are seventeen medical facilities in the area including one referral hospital. This hospital is the only facility providing comprehensive emergency obstetric care (EOC) [15]. The other health facilities provide some basic EOC. General access to basic health care facilities in the country is good, with over 85% of the population living within 3 km of a primary health care or outreach post and 97% within 5 km [16]. The distance from other health facilities in the area to the referral hospital ranges from 17 to 115 km. Antenatal care coverage in the country is exceptionally high, as 96% of pregnant women attend antenatal clinic at least once during pregnancy [17]. Levels of maternal mortality in this region are nevertheless among the highest in the country [7].

### Model

We used the standard WHO definition of maternal death [18]. Like most health problems, causes of maternal death can be viewed either narrowly or broadly. A broad view would take into account individual, community and health service factors that contributed to the deaths, not merely the medical cause. The "Three phases of Delay Model" [19] was chosen to classify factors associated with the maternal deaths in the present study. The model is based on the fact that about 75% of maternal deaths are a result of direct causes: hemorrhage, obstructed labor, sepsis, eclampsia and abortion complications [4]. Most of these deaths are preventable with prompt and adequate medical interventions. Delays in reaching adequate care are prominent factors contributing to maternal deaths. Thaddeus and Maine [19] have argued that not getting adequate care in time is the overwhelming reason why women die in developing countries. Lack of care, they argued, can be related to three factors: a delay in making the decision to seek care when complications develop; a delay in reaching obstetric medical facility once the decision to seek care has been made; or delay in receiving adequate and appropriate care once a medical facility has been reached. Delay in the decision to seek medical care may be influenced by various factors such as the actors involved in the decision-making process, illness characteristics, and experience with the health system or distance to the health facility. Delay in reaching an appropriate medical facility is affected by the distribution of health facilities, availability of transportation, road conditions or cost of transportation. Delay in receiving adequate and appropriate care once the facility is reached is mainly due to operational difficulties in the health care delivery system. Such inadequacies may be characterized by shortages in supplies, equipment, lack of trained personnel, incompetence of the available staff, or uncoordinated emergency services. The delay model helps to identify community and health services factors contributing to maternal deaths and as such it is useful in devising interventions and strategies for preventive measures.

## Methods

### Data Collection

This study is part of an in depth study on maternal mortality in the above described area. The main bulk of maternal mortality studies in the Gambia cover other areas [7-11]. We have, in the chosen time period, identified a total of 42 maternal deaths, by using different community and health facility case finding strategies. The cases identified were women who actually tried to reach or have reached health care services. Medical and social details about the fatal cases are submitted for publication elsewhere. A verbal autopsy technique [20] was possible to perform on 32 of the women who died. We aimed at identifying the various circumstances that contributed to the deaths, often called the "road to death" [21,22]. This involved a visit to the home and village of the deceased and all health facilities where she sought care. Key people who had witnessed any stage in this process were interviewed.

In the community, interviews were performed with family members or other persons, usually those present during the time the deceased developed the illness; or those who accompanied her to the health facilities and were with her at the time of death. These were mainly in-laws, midwives or the deceased's husband. A WHO based, verbal autopsy questionnaire [10] was administered. An interview with the deceased's relatives was performed in the deceased's home at least one week after, but not later than twenty-five weeks after the death.

We often used a group interview, as the whole family participated in revealing their versions of the context of the death, and to give a verbatim account on the deceased's final illness up to her death. Specific issues such as the time taken to decide to seek care; places where care was sought; financial constraints; cultural factors influencing care seeking process; means of transportation and time to reach a medical facility were explored and recorded.

We also interviewed health care staff who participated in the provision of care to the deceased, ambulance drivers, generator operators and laboratory personnel. Other people interviewed in the community were local taxi drivers and ferry captains.

### Analysis

Interviews with staff were carried out independently. All interviews were tape recorded and transcribed in full text. The transcribed material was categorized and analyzed by using a *grounded theory*. Grounded theory is an interview text analysis method utilized for qualitative studies in the health sector, where the basis for the analysis is not a theoretical model per se, but the focus is rather on identified items of relevance to the studied topic [23]. Interpretation of the findings with a view to provide possible and plausible explanations was then performed.

### Ethical approval

The study was approved by a joint ethics committee of The Gambia Government/Medical Research Council Laboratories and the regional Medical Ethics Committee of Norway. Oral consent was taken from each participant.

## Results

### Delay in deciding to seek care

The delay was estimated from the moment somebody, either the woman or her family realized that there was a complication until the decision to seeking care was made. In seven of the 32 cases the process of seeking medical attention was delayed after becoming aware of the complication. The delay ranged from two hours to five days. The reasons mentioned for the delay were underestimation of the severity of the complication, cultural belief or previous unfavorable experience with the health system.

### Underestimation of the severity of the complication

Previous uncomplicated pregnancies may influence actions taken during the current pregnancy that can lead to a delay in the decision-making process. Women or their relatives used previous pregnancies as a risk- predicting tool. A husband of a deceased woman narrated:

"This was her ninth pregnancy. All previous pregnancies were delivered at home. She always gave birth without even calling for help from the traditional birth attendant. We thought she will deliver this time without a problem".

In another case a mother in-law narrated:

"She swept the house and prepared breakfast. At midday she was lying in the room complaining of labor pains. We thought she will deliver without a problem as in her last pregnancy. It was after 2:00 pm she did not deliver then we decided to look for transport to take her to the health centre".

### Cultural belief

In Gambian society pregnancy and childbirth are generally regarded entirely as women's entity. Older women in their menopause are seen as experts on pregnancy and childbirth, particularly in rural areas of the country. These women are consulted if a complication is noticed during pregnancy, labor or during the puerperium. When consulted, they usually decide what should be done and their advice is taken. Words of elders are hardly challenged in Gambian societies. In the case below an older woman advised a woman in labor to wait until after the next Muslim praying time (after three hours) before seeking medical attention. The rationale for the delay was that it is believed to evaluate progress in labor at specific times corresponding to the Muslim praying times:

"Labor and child birth takes place at certain times... ... ....these times correspond with the Muslim praying times. It was around midday and the next praying time was 2:00 pm so we thought she will deliver by then. It was after then when she did not deliver we decided to take her to the health centre".

### Experience with the health care system

Other testimonies indicated that structural factors in maternal health care provision discouraged women from seeking care. Prenatal care is provided on specific days in each community during week days only. This gives the impression that maternal health services are only available on days when clinics are held. A mother in-law narrated:

"On Thursday evening she complained of abdominal pains... ... ...throughout the weekend she was with severe abdominal pain but we had to wait until the following Monday as it is the day on which pregnant women are attended to. The clinic is closed on Saturdays and Sundays".

Poor provider attitude, fear of punishment by health care providers based on previous experiences or just gossip can lead to delays in the decision making process. A midwife narrated

"She was vomiting throughout the night, the following morning the husband decided to take her to the health centre but she refused... ... ...she has not yet get an antenatal care card. She feared the nurses because if she goes to complain about the vomiting she will be asked the card and without it they [nurses] will tell her all salty words. She may be insulted or may even not be given medicine".

Information from care providers that is not clearly understood, can lead to delay in seeking medical care. A woman with twin pregnancy was advised to deliver at the hospital. However, the information provided lead to the following situation:

"She was told she had twin pregnancies by the nurse. She was told by the nurse to report to the hospital when in labor. When labor began we decided to go to the hospital. She was not told to report to the hospital before labor began".

Barriers to seeking care may not appear as such to care providers when they make recommendations. The woman may not see them as an issue, as she is not given room to discuss these with the nurse.

Lack of money and refusal to receive medical attention were not identified as factors affecting health care seeking process. In twenty-two out of the thirty-two women, no funds were available when the complication developed. In all these cases the woman was taken to a medical facility without money and a relative was left behind to raise money in the community, to be able to pay at a later stage.

### Delay in reaching an appropriate medical facility

Once a decision to seek medical care has been made, other obstacles had to be overcome in reaching a medical facility. Twenty-seven of the 32 women were delayed in reaching an appropriate medical facility. The reasons for this delay can be grouped into three subcategories: lack of transport, prolonged transportation and seeking care at more than one facility.

### Lack of transportation

Transportation difficulties, such as poor road conditions, lack of readily available transport and/or inadequate means of transportation were mentioned. The relatives often expressed shortage of transport as serious obstacles. Lack of motorized transport forced some families to opt for alternative means of transport such as using a cart (donkey, ox or horse) or in extreme instances, they walked. A husband explained:

"She started pouring blood late in the evening just after evening prayers [5:00 pm]....we took her to the main road [tarred road] to look for transport. We were there [main road] up to twelve midnight but couldn't get transport. All the vehicles that came were full. We went back home and woke up early morning the following day to catch the first transports".

Transportation difficulties were experienced even after reaching the first medical facility, as some of the health facilities were without an ambulance. If a facility has an ambulance it usually serves multiple purposes and may not be available at certain times. A midwife narrated:

"The patient came to the health centre at around 4:00 pm... ....she cannot be managed here because she may need an operation [caesarean section]. We planned to evacuate her to the hospital but our ambulance had a breakdown a week ago. We looked for transport in the village throughout the night but could not get one. The following morning we went to the agricultural department to look for transport but their vehicle had already left for trek. It returned around 11:00 am and thereafter it came to transport the patient to the hospital".

Lack of fuel for the ambulance was also mentioned. In such occasions relatives or escorts met the fuel cost. A husband narrated:

"I took my wife to the health centre... ...two hours later the nurse told me that she [my wife] will be transferred to the hospital but that the ambulance had no fuel. I was asked to buy fuel for the ambulance to take my wife. I bought twenty liters of diesel".

Some communities in the Gambia have – with the assistance of the health authorities – tried to set up community based emergency transport systems, such as horse carts or bicycle ambulances, but it is difficult to make them sustainable.

### Prolonged transportation

Long distance, visiting different health facilities, poor road and vehicle conditions contributed to prolonged traveling time. Several testimonies highlighted this. A husband explained:

"She was admitted in the hospital for two weeks and discharged on a Monday. On her return to our village [85 km away from the hospital] she fell down unconscious. We took her to the health centre in our village where she was transferred to another health centre [20 km]. She was again transferred to the hospital [60 km away]. She spent few hours at the hospital and died".

### Seeking care at more than one medical facility

Seeking care at an inappropriate level of facility actually delays access to appropriate treatment. The inability to provide comprehensive obstetrical services forces peripheral health facilities to refer all women needing such services to the nearest hospital: 26 of the 32 women visited more than one medical facility during the care seeking process, 18 of the 26 women visited two health facilities while the other 8 women visited three different facilities. Thus, the women accessed a *health care facility*, but not appropriate *health care*. The husband of a deceased narrated:

"We took her to the health centre in the village... .....she was examined by the nurse who later transferred her to another health centre [44 km away]. There she spent the night and the following morning she was again transferred to the hospital [36 km away]. On our way to the hospital we had to cross the river at two different crossing points. Immediately after we reached the hospital she died".

### Delay in receiving prompt and appropriate care after reaching the hospital

Thirty-one women experienced delay in receiving prompt and adequate obstetric care at the hospital level. Lack of blood transfusions and basic medical supplies were mentioned in the testimonies. A mother in-law explained:

"When we reached the hospital, they [the doctor and the nurses] told us to find two bottles of blood for her [our patient]. We went to the laboratory but the man at the lab said there was no blood. I donated one bottle and bought another in the lab. After giving her [patient] the blood we were asked to get another two bottles. We went back to the lab but the man at the lab insisted there was no blood. I paid him D300.00 [equivalent to US$12.00] before getting the two bottles of blood".

A husband of a deceased woman narrated:

"She was pouring blood at home so we took her to the health centre. There we were told she urgently needed blood but blood bags were not available. She was then transferred to the hospital [60 km away]. At the hospital blood bags were finished. She was in the hospital from mid-day up to the following day in the evening but had not received blood. Late at night she died".

A laboratory officer narrated:

"Here patients are escorted to the hospital by old women who are not fit to donate blood. In addition most men in this area are reluctant to donate blood and prefer to buy blood".

Delay in providing prompt and adequate care by the medical team was also highlighted in the testimonies. A midwife narrated:

"She was brought to the hospital on the 13^th ^at around 9:00 am from another health centre. The doctor saw her and diagnosed hand-presentation. He [doctor] asked us [midwives] to observe her. No action was taken by the doctors up to the 15^th ^late in the evening [48 hours later] when they took her to the theatre. He [doctor] first tried external cephalic version which failed before a caesarean section was performed. The patient was wheeled dead from the theatre".

Poor management of staff availability, particularly doctors, has been mentioned as a factor contributing to poor care. A midwife narrated:

"There used to be four doctors in the maternity unit but in July all three went on leave together. Now only one doctor is available for the unit. He does ward rounds, performs operations and runs the out-patients clinic. Even when there were four doctors we usually have problems with them [doctors] because there is no duty rooster for doctors in place. After normal working hours when there is an emergency it is always difficult to see them".

## Discussion

The Gambia was the first country to implement a sisterhood approach to measure maternal mortality rates [9]. Former studies of maternal deaths in the Gambia indicated a decrease in numbers [10]. However, factors related to health care delivery could contribute to further improvement [16], as substandard care has been demonstrated as a contributing factor to poor survival [8]. We used multiple sources of information, such as health workers' identification, community leaders' knowledge, hospital files and post partum follow up visits to identify maternal deaths that took place in the health care facilities.

Maternal death is often a consequence of a long and complex chain of delays, and only in few cases death can be attributed to a specific event [24-26]. Any one delay could be fatal to a woman with obstetrical complications. Contrary to the common belief, that women do not seek care and die in the community, we identified a number of women who initially intended to deliver at home, but tried to get assistance once a complication occurred. The problems encountered in trying to do so, reveal major obstacles in access to appropriate care within an acceptable time.

### Delay in deciding to seek medical care

Delay in deciding to seek medical care on the part of the woman or her relative is usually regarded entirely as patient factor. First, the illness or complication must be recognized and classified as abnormal. Recognition of an illness may be influenced by factors such as the prevalence of the condition [27]. In a study among pregnant women in Senegal, 13% regarded fever, pallor and dizziness as normal signs of pregnancy because these conditions were common among pregnant women in that area [28]. In Tanzania, rural women seem to avoid going to the hospital because of fear of discrimination, geographical and financial barriers and different interpretation of danger signs [29]. Raising awareness is a health education issue for health care workers and the community. One role of appropriate antenatal care is to address these issues and to offer care seeking solutions in advance. Access to skilled attendance at childbirth includes improved technical skills as well as skills in attitude, communication, information and early advice on referral [30].

Brown [31] defined culture as a 'complex whole' that refers to the learnt pattern of thoughts and behavior characteristics of a social group. It involves religion, kinship, knowledge, belief, art, morals and child bearing practices. The tendency to act or not in the presence of a complication is also influenced by the interpretation supported by cultural beliefs. Several studies carried out in Africa and elsewhere [24,32,33] have highlighted how culture influenced health care seeking process. Religious belief was mentioned to have influenced the care seeking process in our study. Jansen [34] asserted that religion, medicine and magic are closely interwoven. If the barriers to care are too overwhelming, a culturally based reassurance that "things most likely will go well" may cause a hesitation in recognizing early signs of complications.

Health service related factors were mentioned to have constrained the decision-making process in this study. Bad experience with the health system will mostly lead to reluctance or non-utilization of health care services. Poor provider attitude towards patients has been identified as a major factor to low utilization of services in Kigoma [21] and to low compliance to a referral hospital by high-risk pregnant women [28]. The communication barriers between lay people's concepts and those of professional care providers may lead to serious misinterpretations. Women in the Gambia often resort to home delivery assisted by a traditional birth attendant or a relative as their first option. Sundari [25] identified unfamiliar setting at the health facility, being attended to by strangers, lack of family support, attendant being a male care provider, reduced autonomy, lack of sympathy and understanding on the part of the health care personnel and not seeing the need for care as some of the factors contributing to non-utilization of health services during labor and childbirth.

### Delay in reaching an appropriate medical facility

Lack of public transportation systems in rural areas requires that communities need to form partnership with the local commercial transport owners in addressing the transport problem. This strategy was adopted in North-western Nigeria [35] and had contributed to the reduction of maternal deaths and cost of transportation.

Major health centers are strategically located in the Gambia, but accessing them does not necessarily mean to receive appropriate care. Sometimes using these sites as the entry point to health services can delay further attempts of accessing adequate care. Efforts to transfer health centers into fully functional basic obstetric emergency units could reduce the delay caused by long transportation time.

Being unable to meet the costs for immediate health care was not seen as a main obstacle. Some health facilities supported by non-governmental organizations (NGOs) and local associations have implemented a system of cost sharing in order to provide quality health care.

### Delay in receiving prompt and appropriate care after reaching the hospital

A multi-centre study from three West-African countries, reported that most of the women classified as "near misses" were referred from another facility [36], highlighting the need to differentiate between those who arrive in a critical condition and those who develop one. Inadequacy in health care may be due to one or a chain of the following events: shortage of medical supplies, lack of equipment, lack of trained personnel, and incompetence of the available staff. Health system failures have been identified as a major contributing factor to maternal deaths [8,10,21,37,38].

## Conclusion

The failure to get adequate treatment in time may be seen in a "right to access health care "context. Women's access to appropriate services is a concern in the Gambia. This study reveals that women do try to reach adequate health services when an emergency occurs, but that there are many obstacles that delay this process. Improving accessibility and quality of EOC services in the area is necessary if maternal deaths are to be prevented

## Competing interests

There are no competing interests for this study. The study was partly financed by a Norwegian government quota grant for students from developing countries for higher studies in Norway. This project was also funded by the Participatory Health Population and Nutrition Project (PHPNP) of the Department of State for Health of The Gambia.

## Authors' contributions

Mamady Cham did the data collection in the field, the main analysis, wrote the first draft of the paper and reviewed the final document.

Johanne Sundby planned the current study and introduced the methodology, and participated in the writing of the first draft, and wrote the final version of the document.

Siri Vangen assisted in the analysis of data as the second reviewer of the text, contributed to the first writing of the paper and reviewed the final document.
